# The Cassava Mealybug (*Phenacoccus manihoti*) in Asia: First Records, Potential Distribution, and an Identification Key

**DOI:** 10.1371/journal.pone.0047675

**Published:** 2012-10-15

**Authors:** Soroush Parsa, Takumasa Kondo, Amporn Winotai

**Affiliations:** 1 Centro Internacional de Agricultura Tropical (CIAT), Cali, Colombia; 2 Corporación Colombiana de Investigación Agropecuaria (CORPOICA), Centro de Investigación Palmira, Palmira, Valle, Colombia; 3 Entomology and Zoology Group, Plant Protection Research and Development Office, Department of Agriculture, Chatuchak, Bangkok, Thailand; Roehampton University, United Kingdom

## Abstract

*Phenacoccus manihoti* Matile-Ferrero (Hemiptera: Pseudococcidae), one of the most serious pests of cassava worldwide, has recently reached Asia, raising significant concern over its potential spread throughout the region. To support management decisions, this article reports recent distribution records, and estimates the climatic suitability for its regional spread using a CLIMEX distribution model. The article also presents a taxonomic key that separates *P. manihoti* from all other mealybug species associated with the genus *Manihot*. Model predictions suggest *P. manihoti* imposes an important, yet differential, threat to cassava production in Asia. Predicted risk is most acute in the southern end of Karnataka in India, the eastern end of the Ninh Thuan province in Vietnam, and in most of West Timor in Indonesia. The model also suggests *P. manihoti* is likely to be limited by cold stress across Vietnam's northern regions and in the entire Guangxi province in China, and by high rainfall across the wet tropics in Indonesia and the Philippines. Predictions should be particularly important to guide management decisions for high risk areas where *P. manihoti* is absent (e.g., India), or where it has established but populations remain small and localized (e.g., South Vietnam). Results from this article should help decision-makers assess site-specific risk of invasion, and develop proportional prevention and surveillance programs for early detection and rapid response.

## Introduction

**Figure 1:**
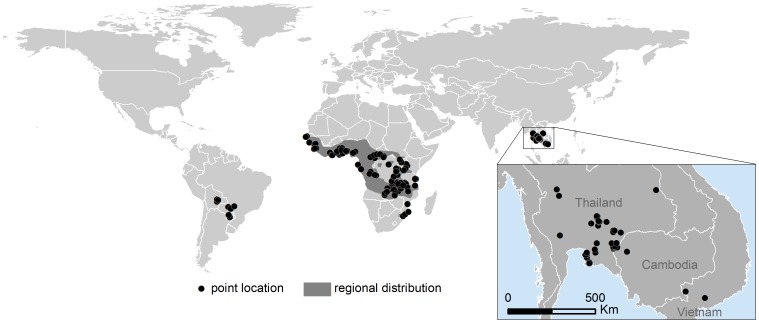
Known distribution of *Phenacoccus manihoti*. Point locations in South America correspond to its native distribution, and were georeferenced from figure 1 in Löhr and Varela [2]. Regional distribution in Africa was adapted from figure 1 in Herren and Neuenschwander [30]. Point locations in Africa correspond to *Anagyrus lopezi* releases at locations with high *P. manihoti* infestations, and were georeferenced from Neuenschwander [3]. Point locations in Asia correspond to reports listed on Table 1.

The cassava mealybug, *Phenacoccus manihoti* Matile-Ferrero (Hemiptera: Pseudococcidae), is one of the most severe pests of cassava (*Manihot esculenta*) in the world [1]. It is native to South America [2], but it has become naturalized throughout sub-Saharan Africa since its inadvertent introduction into the continent in the early 1970s (Fig. 1) [3]. *P. manihoti* was not known to occur in Asia until 2008, when it was first detected in Thailand. Since that year, it has spread aggressively throughout Thailand's cassava-growing region [4], also invading its neighboring countries and Indonesia [5] (Fig. 1), and raising significant concern over its potential arrival to more countries [6]. Responding to this concern, we present the first records of *P. manihoti* invading Asia and use them to estimate the climatic suitability for its establishment throughout the region. To further support detection and response efforts, we also provide a taxonomic key that differentiates all mealybug species recorded from the genus *Manihot*.

**Figure 2:**
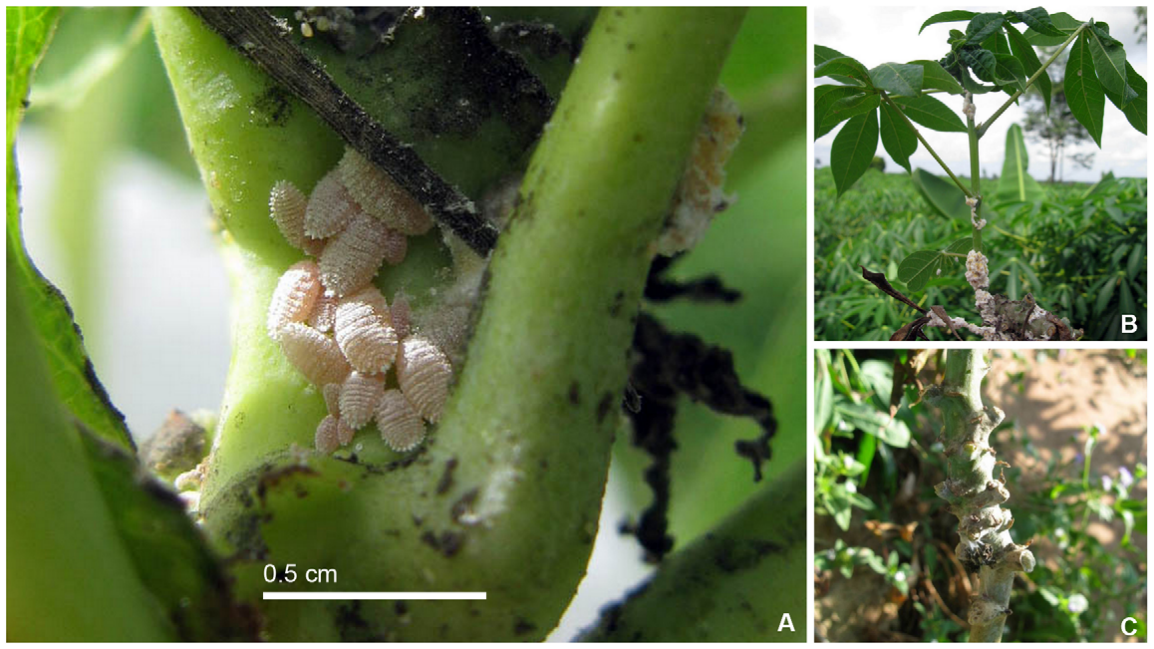
*P. manihoti* infestations and associated symptoms on cassava in Asia. A. *P. manihoti* populations at upper nodes. B. Heavy infestations and associated terminal shoot distortion. C. Stem deformation associated with *P. manihoti* infestations. Photo credits: A and B: S. Parsa; C: A. Winotai.

Several non-preferred host species can support *P. manihoti* reproduction, but only cassava is known to experience significant damage by this insect [7]. When it feeds on cassava, *P. manihoti* causes severe distortion of terminal shoots, yellowing and curling of leaves, reduced internodes, stunting, and weakening of stems used for crop propagation (Fig. 2). In the absence of its natural enemies and other control measures, this damage can reduce yields by more than 80% [8]. No cassava cultivars are known to be fully resistant to *P. manihoti* [9]. Explorations for *P. manihoti* natural enemies within its native range identified four hymenopterous parasitoids, twelve predators and one entomopathogenic fungus [2], [10] out of which the parasitoid *Anagyrus lopezi* appeared to be one of the most promising [2]. The introduction of this parasitoid into Africa in the 1980s reduced high infestations by 90%, becoming a highly-successful case of classical biological control [11]–[13]. A similar outcome is expected from its recent introduction to Thailand, in November 2009 [4].

*P. manihoti* is parthenogenic, producing only female offspring. Hence, a single immature or adult may be sufficient to start an outbreak. Under optimal conditions, adults can deposit between 200–600 eggs [14], [15] within ovisacs on the undersides of leaves and around apical and lateral buds. Ovisacs are sticky and can adhere to clothing, facilitating long-distance mealybug dispersal. Eggs hatch into mobile crawlers that can spread over the plant or be passively dispersed to neighboring plants by wind. Crawlers commence feeding from phloem fluids in young leaves and stems, and pass through three nymphal instars before reaching maturity. Under laboratory conditions at 25°C, egg to adult development takes an average of 31–33 days [16], [17]. Development is optimal around 27°C [15], and significant mortality occurs below 15°C [10] and above 33°C [16], [18]. Rainfall is a key determinant of *P. manihoti* abundance and population dynamics: dry regions, years and seasons favor outbreaks [19], [20]. Rainfall is thought to suppress *P. manihoti* mainly by causing mechanical mortality [21], but also by favoring insect pathogens and reducing cassava's suitability as a host [9], [22].

Pest risk maps, based on models predicting climatic suitability for a species, are important decision-support tools for the management of invasive pests like *P. manihoti* [23]. Two modeling approaches are often used to develop them. The correlative or inductive approach estimates a species' climatic preferences based on analyses of geographic occurrence data [24]. By contrast, the mechanistic or deductive approach estimates its climatic preferences based on laboratory experiments [25]. Outputs from correlative models often align more closely with known distributions without demanding any biological data, but mechanistic models are thought to be superior in predicting distributions in novel environments [26]. Hence, integrative approaches that draw upon the complementary strengths of both can provide a very good approximation to the potential distribution of an invasive pest [27]–[29]. In this article, we use an integrative modeling approach to predict *P. manihoti*'s potential distribution in Asia, in order to support decision-making in the management of this invasive pest.

**Table 1:** Georeferenced reports of *Phenacoccus manihoti* on cassava in Asia (2008–2012).

Country	Province	Lat	Long	Determined by	Date
**Thailand**	**Rayong**	**12.731**	**101.139**	**D.J. Williams**	**07-Oct-08**
**Thailand**	**Chachoengsao**	**13.762**	**101.512**	**P. Hernandez**	**13-Oct-08**
Thailand	Rayong	12.734	101.136	G. Watson	02-Nov-08
Thailand	Nakhon Ratchasima	14.669	101.563	G. Watson	20-Nov-08
**Thailand**	**Rayong**	**12.731**	**101.139**	**A. Winotai**	**08-Jan-09**
Thailand	Chon Buri	13.034	100.997	A. Winotai	08-Jan-09
Thailand	Chon Buri	13.185	100.997	A. Winotai	15-Feb-09
**Thailand**	**Mukdahan**	**16.491**	**104.562**	**D.J. Williams**	**18-Feb-09**
**Thailand**	**Chon Buri**	**13.174**	**100.931**	**D.J. Williams**	**24-Feb-09**
Thailand	Nakhon Ratchasima	14.915	101.599	A. Winotai	13-Aug-09
Thailand	Chon buri	13.284	100.992	A. Winotai	20-Aug-09
Thailand	Nakhon Ratchasima	14.858	102.015	A. Winotai	03-Sep-09
Thailand	Saraburi	14.776	101.224	A. Winotai	16-Sep-09
Thailand	Nakhon Ratchasima	15.157	101.502	A. Winotai	16-Sep-09
Thailand	Kanchanaburiá	14.168	99.618	A. Winotai	26-Sep-09
Thailand	Nakhon Ratchasima	14.411	102.359	A. Winotai	08-Oct-09
Thailand	Chon Buriá	13.441	101.405	A. Winotai	10-Nov-09
**Thailand**	**Chon Buri**	**13.279**	**101.440**	**D.T. Kondo**	**10-Nov-09**
**Thailand**	**Rayong**	**12.731**	**101.139**	**P. Hernandez**	**10-Nov-09**
**Thailand**	**Buri Ram**	**14.314**	**102.741**	**P. Hernandez**	**11-Nov-09**
**Thailand**	**Nakhon Ratchasima**	**14.416**	**102.421**	**P. Hernandez**	**11-Nov-09**
**Thailand**	**Nakhon Ratchasima**	**14.854**	**101.612**	**P. Hernandez**	**11-Nov-09**
Thailand	Buri Ram	14.314	102.741	P. Hernandez	11-Nov-09
**Thailand**	**Nakhon Ratchasima**	**15.114**	**101.504**	**P. Hernandez**	**12-Nov-09**
Thailand	Kamphaeng Phet	16.527	99.446	A. Winotai	16-Mar-10
Thailand	Kamphaeng Phet	16.202	99.562	A. Winotai	18-Mar-10
Thailand	Kamphaeng Phet	16.530	99.453	A. Winotai	18-Mar-10
Cambodia	Battambang	13.327	103.061	A.C. Bellotti	24-Jul-10
Cambodia	Banteay Meanchey	13.490	102.371	A.C. Bellotti	25-Jul-10
**Cambodia**	**Banteay Meanchey**	**13.549**	**102.559**	**P. Hernandez**	**25-Jul-10**
Thailand	Sa Kaeo	13.600	102.445	A.C. Bellotti	26-Jul-10
Thailand	Sa Kaeo	13.758	102.279	A.C. Bellotti	26-Jul-10
Thailand	Sa Kaeo	13.773	102.525	A.C. Bellotti	26-Jul-10
**Thailand**	**Sa Kaeo**	**13.758**	**102.279**	**P. Hernandez**	**26-Jul-10**
**Vietnam**	**Tay Ninh**	**11.288**	**106.078**	**P. Hernandez**	**06-Jan-12**
Vietnam	Dong Nai	10.947	107.076	T. Aye	28-Feb-12

*Notes*: Reports in bold correspond to specimens studied to verify identification key and were deposited at the entomological reference collection of the International Center for Tropical Agriculture (CIAT).

## Materials and Methods

### Known distribution map

*P. manihoti*'s distribution records in South America and Africa were obtained from the published literature. Native distribution records were obtained from Löhr et al. [2]. Naturalized distribution records in Africa correspond to *A. lopezi* release sites [3], presumably at locations with high *P. manihoti* infestations, and to a regional distribution map adapted from Herren and Neuenschwander [30]. Geographic coordinates were approximated either by georeferencing published maps or by searching locations on Google Maps or on the MarkSim shape file for African towns (afrtowns.shp) [31]. Invasive distribution records in Asia correspond to specimens either collected by or submitted to the authors for identification. The first specimens were submitted for authoritative taxonomic identification to experts Dr. Gillian Watson (Department of Food and Agriculture, California) and Dr. Douglas J. Williams (Natural History Museum, London). Subsequent identifications were verified by TK and/or Maria del Pilar Hernandez (see reports in bold on Table 1). These latter specimens are deposited at the CIAT Entomology Collection, Palmira, Colombia. Invasive distribution records were georeferenced using geographic positioning system (GPS) receivers (GPSMap 76CSx, Garmin Ltd., Olathe, KS). The map was developed using geographic information system (GIS) software (ArcGIS, ESRI, Redlands, CA).

**Figure 3:**
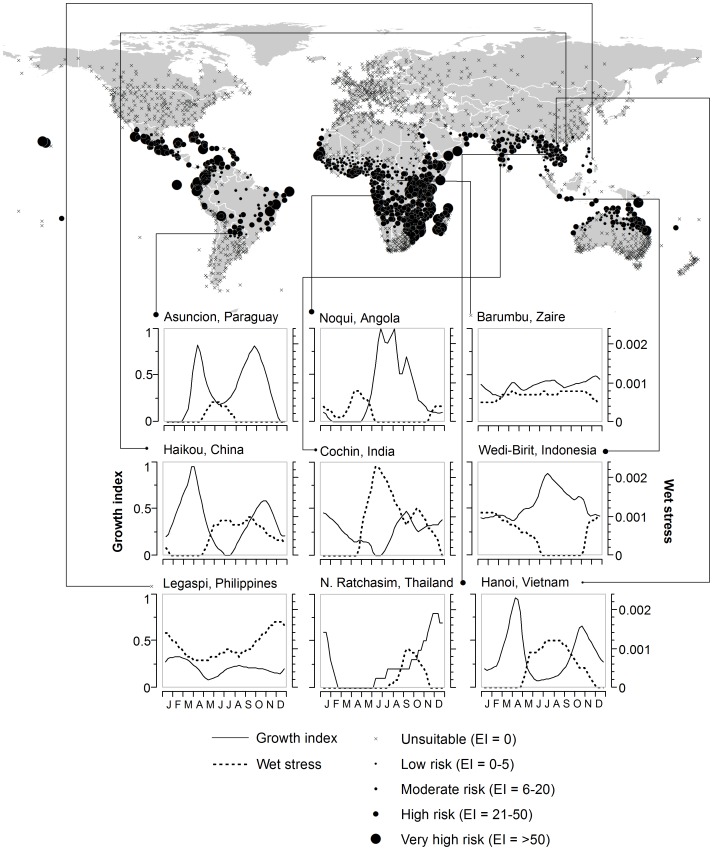
CLIMEX climatic suitability indices for *Phenacoccus manihoti* at selected locations. Predictions are based on the ecological index (EI), a measure of climatic suitability scaled from 1–100, for locations within CLIMEX's station database. The growth index captures conditions suitable for population growth and the wet stress captures mortality due to rainfall only. Predictions for Asuncion and Noqui correlate with *P. manihoti* seasonal dynamics reported by Löhr and Varela [2] and Leuschner and Nwanze [39] respectively.

**Table 2:** Parameter values used to build a predictive distribution model for *Phenacoccus manihoti* in CLIMEX.

Description	Parameter	Value	Unit
**Population growth: temperature**			
Lower temperature threshold	DV0	16	°C
Lower optimum temperature	DV1	24	°C
Upper optimum temperature	DV2	29	°C
Upper temperature threshold	DV3	34	°C
Development (egg-adult) heat demand	PDD	290	°C days
**Population growth: moisture**			
Lower soil moisture threshold	SM0	0	SMC^1^
Lower optimum soil moisture	SM1	0.01	SMC
Upper optimum soil moisture	SM2	0.5	SMC
Upper threshold of soil moisture	SM3	2.5	SMC
**Mortality: temperature**			
Lower developmental temperature threshold	DVCS	16	°C
Weekly degree-day threshold for cold stress	DTCS	21	°C days
Cold stress accumulation rate	DHCS	−0.0015	week^−1^
Weekly heat stress temperature threshold	TTHS	35	°C
Heat stress accumulation rate	THHS	0.001	week^−1^
**Mortality: moisture**			
Weekly wet stress soil moisture threshold	SMWS	0.8	SMC
Wet stress accumulation rate	HWS	0.00125	week^−1^

1 Proportion of soil water holding capacity.

### Potential distribution model

We modeled *P. manihoti*'s potential distribution using CLIMEX Version 3 [32], a software widely used with positive results in the fields of biological control, pest risk assessment and climate change [33], [34]. The CLIMEX Compare Locations module uses an integrative inductive-deductive approach to estimate climatic suitability for a species based on both (1) geographic occurrence data and on (2) the species' growth response under experimentally-manipulated conditions [32], [35]. Climatic suitability is estimated by the ecoclimatic index (EI). The EI reflects the annual balance between population growth during favorable seasons (captured by the annual growth index [GI_A_]) and mortality during unfavorable seasons (captured by stress indices for cold [CS], heat [HS], drought [DS] and wetness [WS]) [35]). Theoretically, EI is scaled between 0 and 100, and the larger the EI the more suitable the location for the species. In practice, EI values below 10 indicate marginal suitability, EI values above 20 indicate high suitability, and EI values above 50 are rare and usually confined to the tropics [33]. Formulas governing the CLIMEX Compare Locations model have been published by Sutherst and Maywald [35].

**Figure 4:**
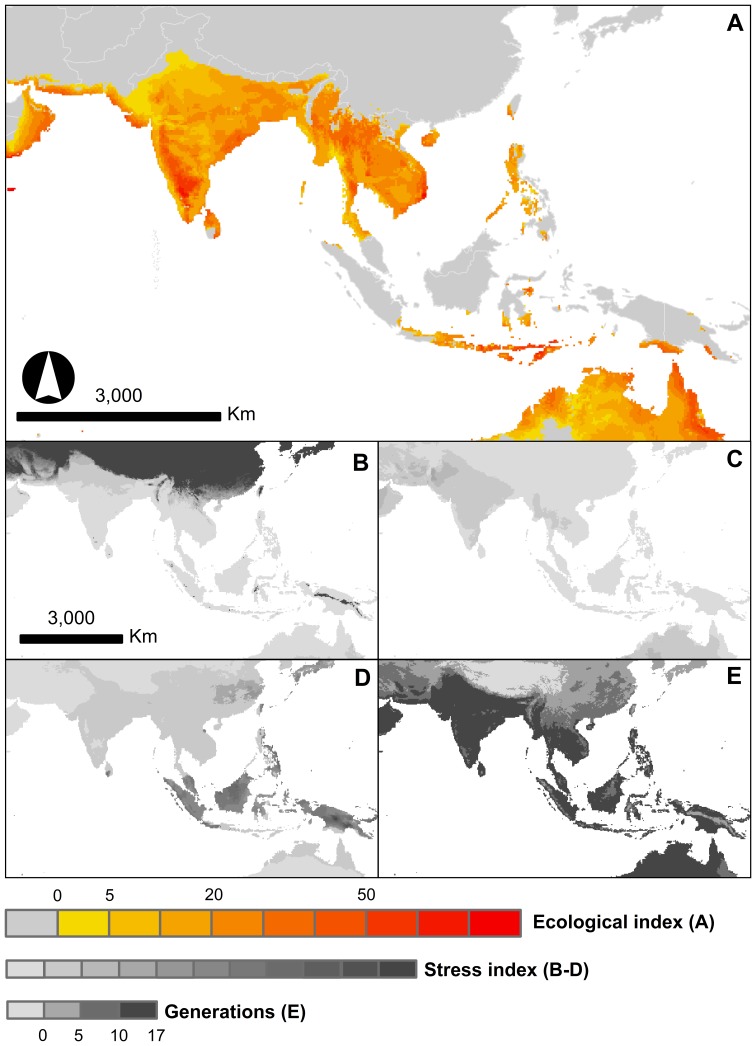
CLIMEX climatic suitability indices for *Phenacoccus manihoti* in Asia. Predictions used the CLIMOND interpolated climate database at 10′. A. Ecological index (EI), a measure of climatic suitability from 1–100. EI values greater than 20 indicate high risk of infestations. B. Cold stress, an index of mortality caused by intolerable cold. C. Heat stress, an index of mortality caused by intolerable heat. D. Wet stress, an index of mortality caused by rainfall. E. Number of generations per year *P. manihoti* can potentially complete at a given location.

### Climate data

CLIMEX models demand weekly, temporally-interpolated data from averages of five variables: maximum and minimum temperatures, 9 a.m. and 3 p.m. relative humidity, and rainfall (i.e., 260 data points per location). We used two metereological databases to provide this data. To streamline model development iterations, we first used the less computationally-demanding station database built into CLIMEX. This is a point location database with records from about 2,400 metereological stations worldwide. We then used CliMond 10′ interpolated climate database for CLIMEX [36] to project model results globally.

**Figure 5:**
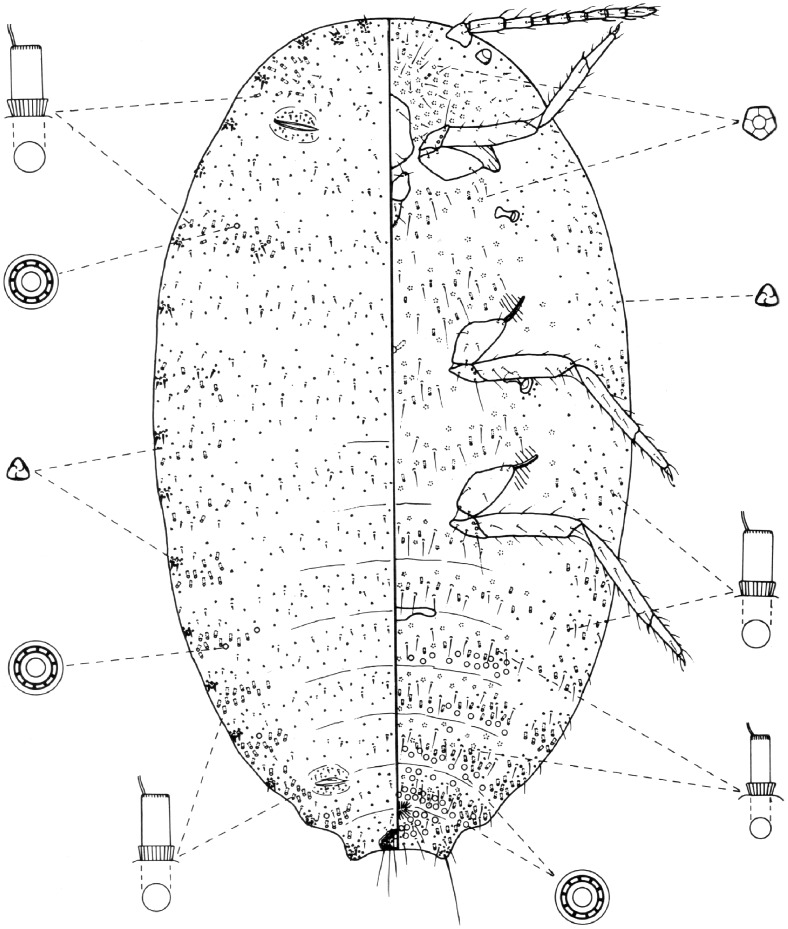
*Phenacoccus manihoti* Matile-Ferrero, adult female. Illustration after Cox & Williams [45], with modification.

### Model fitting

#### Population growth parameters

We used eight parameters to define conditions suitable for *P. manihoti* population growth. Four parameters (DV0-DV3) captured the temperature optima and bounds for growth. An initial range of values for these parameters were obtained from reviewing published experimental studies on *P. manihoti* development [14]–[18], [37]. Four additional parameters (SM0–SM3) captured the moisture optima and bounds for growth, in proportional units of soil water holding capacity. Values for these parameters were assigned under the assumption that *P. manihoti* growth is not directly limited by moisture, but it is optimal when its host is under drought stress [22]. We used a final parameter (PPD) to denote the degree days above the lower threshold for development (DV0) needed by *P. manihoti* to complete one generation. This parameter was used to estimate the potential number of generations *P. manihoti* can complete in one year at a given location. Parameter values for population growth were assigned so as to allow stress indices to explain a greater proportion of EI. For example, we set the upper threshold of soil moisture (SM3) at 2.5, fully aware that mortality by rainfall probably begins at much lower soil moisture levels.

**Figure 6:**
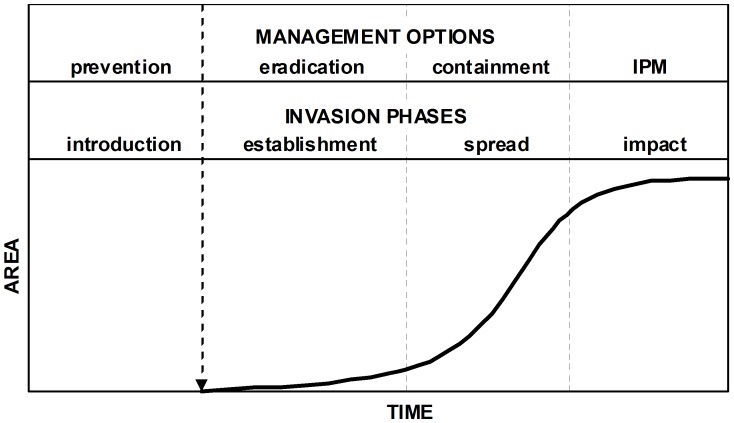
Relationship between successive invasion phases and corresponding management options.

#### Mortality or “stress” parameters

After parametrizing population growth, we used seven mortality parameters in a stepwise inductive process to confine the predicted distribution of *P. manihoti*, reconciling predictions with known distribution patterns in Figure 1. Three parameters captured mortality due to extreme cold, limiting sub-tropical distributions in South America and Africa without affecting distributions in northern Thailand. Parameter values were adjusted such that cold stress (CS) accumulates at a rate (DHCS) of −0.0015 week^−1^ when the total weekly number of degree days above a threshold (DVCS) of 16°C is below the cold stress threshold (DTCS) of 21°C days. These values are conservative, rendering a location unsuitable for *P. manihoti* only after eight consecutive weeks at an average weekly minimum of 15°C, a temperature leading to very high *P. manihoti* mortality in the laboratory [10]. Two parameters captured mortality due to extreme heat, mainly limiting distributions in the African Sahel. Parameter values were adjusted such that heat stress (HS) accumulates at a rate (THHS) of 0.001 week^−1^ when the average weekly maximum temperature is above the heat stress threshold (TTHS) 35°C. These values are also conservative relative to laboratory experiments, which suggest *P. manihoti* cannot survive prolonged periods at or above 33°C [16]. Finally, two parameters captured mortality due to rainfall; limiting distributions in the Congo Basin but not in the west, south and southwest of the Democratic Republic of the Congo (previously Zaire); thereby approximating mealybug distribution maps for that country [38]. Parameter values were adjusted such that wet stress (WS) accumulates at a rate (HWS) of 0.00125 week^−1^ when soil moisture is above the threshold (SMWS) of 80% water holding capacity.

#### Model validation

We validated our model qualitatively, by evaluating the ability of its weekly output indices for population growth (GI) and rainfall mortality (WS) to match *P. manihoti* seasonal population dynamics observed at specific locations in Paraguay [2] and in the Democratic Republic of the Congo [39]. For this evaluation, we selected the locations within the CLIMEX station database that were closest to the study sites.

### Description and identification key

According to the scale insect database ScaleNet [40] there are currently 26 mealybug species (Hemiptera: Pseudococcidae) recorded on the genus *Manihot*, of which 23 have been recorded on cassava, *Manihot esculenta* (Euphorbiaceae). An additional species, *Phenacoccus solenopsis* Tinsley, not listed in ScaleNet, has been reported on cassava [41], increasing the number of species recorded on cassava to 24 and 27 on the genus *Manihot*.

In order to facilitate the identification of mealybugs that may be found on cassava, TK prepared a taxonomic key that differentiates all mealybug species hitherto recorded from the genus *Manihot* worldwide. Morphological features of mealybugs needed to prepare the key were taken from descriptions by Williams [42] and Williams & Granara de Willink [43], and the key was constructed mainly by adapting the keys to mealybugs by Williams & Granara de Willink [43]. The key should be used by a trained person or by a specialist since basic knowledge on the morphology of Pseudococcidae is needed in order to interpret the different morphological features used in the key. There is always a possibility that a species not included in the key may be found feeding on cassava, thus the following key should be used with caution.

## Results

### Known distribution map

*P. manihoti*'s native distribution in South America, naturalized distribution in Africa and invasive distribution in Asia is presented in Figure 1. Distribution points in Asia correspond to reports listed in Table 1. The first authoritatively-verified specimens of *P. manihoti* from Asia were collected between October and November of 2008. The distribution map was not intended to be comprehensive, but rather to capture sufficient environmental heterogeneity to guide model parametrization.

### Potential distribution model

Parameter values for the *P. manihoti* distribution model are presented in Table 2. Spatio-temporal predictions for locations within CLIMEX's station database are shown in Figure 3. Spatial predictions adequately match the known distribution map for *P. manihoti* in South America, Africa and Asia. Weekly growth (GI) and wet stress (WS) indices for Asuncion (Paraguay) and Noqui (Angola) match *P. manihoti* seasonal population dynamics at nearby locations [2], [39]. Specifically, the model adequately predicts population peaks from August-November around Asuncion [2], and from June to October around Noqui [39]. Weekly indices at Barumbu (Zaire) explain the unsuitability of the Congo Basin for *P. manihoti*.

Predictions for Asia based on CliMond 10′ interpolated climate database are shown in Figure 4. All distribution records in Asia fall within predicted suitable regions, mostly within regions predicted to be at high risk of outbreaks (EI>20). The highest predicted suitability within cassava-growing regions in Asia is found within the southern end of Karnataka in India, the eastern end of the Ninh Thuan province in Vietnam, and in most of West Timor in Indonesia. Cold stress (CS) explains predicted unsuitability across Vietnam's northern regions and in the entire Guangxi province in China (Fig. 4B). Also, wet stress (WS), or rainfall mortality, explains predicted unsuitability across much of the wet tropics in Indonesia and the Philippines (Fig. 4D). The model also suggests that in Asia *P. manihoti* is not limited by heat stress (Fig. 4C) and can potentially complete up to 17 generations in one year (Fig. 4E).

### *Phenacoccus manihoti* Matile-Ferrero

*Phenacoccus manihoti* Matile-Ferrero, 1977: 146 [44]; Cox & Williams, 1981: 253 [45]; Williams *et* *al*., 1981: 88 [46].

#### Materials examined

Specimens used to verify the key are reported in bold in Table 1.

#### Diagnosis

In life, species pinkish, covered in a white mealy secretion, with tufts of flocculent waxy secretion at posterior end and around margins (Fig. 2A). The species always reproduces parthenogenetically. The species is most similar in life to *Phenacoccus herreni* Cox & Williams which is yellowish and reproduces bi-parentally.

Slide preparations remarkably similar to those of *P. herreni* Cox & Williams. *Phenacoccus manihoti* possess 18 pairs of cerarii (Fig. 5), each with two enlarged lanceolate setae; the dorsal setae are minute and lanceolate; without aggregations of trilocular pores around the setal collars. Quinquelocular pores are numerous on the venter as in *P. herreni*, but there are always 32–68 on the head in the area immediately anterior to the clypeolabral shield, whereas *P. herreni* has 0–20 in this area. Normally the dorsal multilocular pores around the margins are more numerous in *P. manihoti* than in *P. herreni*. They are sometimes present on the thorax in *P. manihoti* but have not been observed in *P. herreni*. Groups of tubular ducts are present around the dorsal margins as in *P. herreni*, although in most specimens they are more numerous in *P. manihoti*. Other important features are the 9-segmented antennae, denticles on the claws and a circulus that is distinctly ‘ox-yoked’ shaped (diagnosis adapted from reference [43]).

An illustration of *P. manihoti*, adapted with permission from Cox & D.J. Williams [45], is presented in Figure 5. The number of multilocular disc-pores on the head region was increased in order to match the minimum number of these pores in this area. No other changes were made to the illustration.

### Key to mealybugs (Hemiptera: Pseudococcidae) recorded on Manihot spp. (Euphorbiaceae) in the World [Adapted from Williams [42] and Williams & Granara de Willink [43]

1. Trilocular pores absent …………………………………….…………………………………… ***Hypogeococcus spinosus*** Ferris- Trilocular pores present …………………………………2

2. Dorsal tubular ducts large, each with orifice surrounded by a round, sclerotized area containing 1 seta or more within its borders, or with the setae adjacent to the rim…………….…3- Dorsal tubular ducts, if present, without this combination of characters………………………………………………………... 6

3. Multilocular disc pores absent entirely. Dorsal ducts each with some setae within the sclerotized rim and other ducts each with some setae outside the border of the rim………………………………………………***Ferrisia meridionalis*** Williams- Multilocular disc pores present. Dorsal ducts not variable, either with setae within the sclerotized rim or all outside the border of the rim…………………………………………….………………… 4

4. Multilocular disc pores present in a row on abdominal segment VI……………………………………………………… …………………………………… ***Ferrisiavirgata*** (Cockerell)- Multilocular disc pores absent from abdominal segment VI, present around vulva only……………………………………… 5

5. Setae associated with sclerotized rim of each dorsal duct situated just outside the border of the rim ……………………………………………………………***Ferrisia malvastra*** (McDaniel)- Setae associated with sclerotized rim of each dorsal duct situated just inside the border of the rim ……………………………………………………… ***Ferrisia terani*** Williams & Granara de Willink.

6. Dorsal surface with setae, on posterior segments at least, broadly lanceolate and same size and shape as posterior cerarian setae ……………………………………………………..……… 7- Dorsal setae with all setae flagellate, normally much thinner than cerarian setae…………………………………………………… 8

7. Ventral multilocular disc pores present on abdomen only, in medial areas, not reaching margins. Oral collar tubular ducts few, present medially or submedially only…………………………………………………………………***Nipaecoccs nipae*** (Maskell)- Ventral multilocular disc pores and oral collar tubular ducts numerous, reaching thorax, present medially and in marginal zone ……………….……………… ***Nipaecoccus viridis*** (Newstead).

8. Cerarii anterior to anal lobe pair, mostly with auxiliary setae ……………………….………………………………………… 9- Cerarii anterior to anal lobe pair, without auxiliary setae …… 16

9. Oral rim tubular ducts completely absent ………………10- Oral rim tubular ducts present ……………………………… 11

10. Dorsal setae on segments VII and VIII conspicuously longer than remaining dorsal setae. Discoidal pores normally present next to eyes ……………………***Dysmicoccus brevipes*** (Cockerell)-Dorsal setae on segments VII and VIII normally not longer than remaining dorsal setae. Discoidal pores normally absent next to eyes ……………………………***Dysmicoccus texensis*** (Tinsley)

11. Oral rim tubular ducts numbering 2 or 3 present next to each of most cerarii, each group with 1 duct larger than others. Multilocular disc pores on venter present only around vulva … ……...…………………………………………….***Pseudococcus longispinus*** (Targioni Tozzetti)-Oral rim tubular ducts next to any cerarius absent or present singly, of 1 size only. Multilocular disc pores on venter present at least as far forward as abdominal segment IV ……………………………………………………. 12

12. Discoidal pores next to each eye present in a sclerotized rim ………………………………………………………………… 13- Discoidal pores next to each eye not present in a sclerotized rim …………………………………………………………….. 14

13. Translucent pores present on hind femora as well as on hind tibiae. Oral rim tubular ducts present on dorsum. Oral collar tubular ducts on mesothorax, opposite each anterior spiracle (cerarius 12), numbering about 15………………………………………………***Pseudococcus jackbeardsleyi*** Gimpel & Miller- Translucent pores absent from hind femora, present on hind tibiae. Oral rim tubular ducts usually absent from dorsum. Oral collar tubular ducts on mesothorax, opposite each anterior spiracle (cerarius 12), numbering about 4 …………...……………………………………………… ***Pseudococcus landoi*** (Balachowsky).

14. Dorsal oral rim ducts rarely numbering more than 5, absent medially on abdomen. Oral collar tubular ducts present submarginally on dorsum of abdomen, absent from margins………………………………… ***Pseudococcus mandio*** Williams- Dorsal oral rim ducts numbering considerably more than 5, at least some present medially on abdomen. Oral collar tubular ducts absent submarginally from dorsum of abdomen, present on margins ………………………………………………………………… 15

15. Normally with an oral rim tubular duct present above each anterior ostiole, next to each postocular cerarius. Ventral marginal oral collar tubular ducts opposite each mid-coxa usually numbering more than 5 …………***Pseudococcus maritimus*** (Ehrhorn)- Normally without an oral rim tubular duct present above each anterior ostiole, next to each postocular cerarius. Ventral marginal oral collar tubular ducts opposite each mid-coxa usually numbering fewer than 5 .……………***Pseudococcus viburni*** (Signoret).

16. Cerarii numbering no more than 6 pairs, present on abdomen only, except for frontal cerarii ocassionally present ………………………… ***Maconellicoccus hirsutus*** (Green)- Cerarii numbering 9–18 pairs; present on abdomen and at least on thorax ……………..……. 17.

17. Oral rim tubular ducts, each with well-developed rim …………………..……………………………………………... 18- Oral rim tubular ducts entirely absent ……………………… 19

18. Dorsal oral rim tubular ducts present on margins only ………… ***Paracoccus marginatus*** Williams & Granara de Willink- Dorsal oral rim tubular ducts present in rows across the segments ……………… ***Paracoccus herreni*** Williams & Granara de Willink.

19. Anal lobe bars present. Antennae with 8 segments. Claw denticles absent ……………………………………………… 20- Anal lobe bars absent. Antennae with 9 segments. Claw denticles usually present, although they may be barely perceptible ……………………………………………………………………… 23

20. Venter of head with 0–35 oral collar tubular ducts. Longest dorsal setae on median area of abdominal segment VI or VII 13–33 μm long. Cerarian setae on head and thorax always conical. Translucent pores never present on hind femora. Median ventral area of abdominal segment VII with single or double row of multilocular disc pores ………………………………………… 21- Venter of head with 0–4 oral collar tubular ducts. Longest dorsal setae on median area of abdominal segment VI or VII 25–50 μm long. Cerarian setae on head and thorax often long and slender. Translucent pores often present on hind femora. Median ventral area of abdominal segment VII with single row of multilocular disc pores………………… ***Planococcus halli*** Ezzat & McConnell.

21. Venter of head with 14-35 tubular ducts and/or thorax with 7–30 ducts near eighth pair of cerarii. Ducts on head and next to eighth pair of cerarii totaling 15–50.…***Planococcus citri*** (Risso)- Venter of head with 0–13 tubular ducts. Thorax with 0–6 ducts next to eighth pair of cerarii. Ducts on head and next to eight pair of cerarii totaling 0–18 …………………………………………22

22. Ratio of lengths of hind tibia + tarsus to trochanter + femur 1.05–1.15. Multilocular disc pores on posterior edges of abdominal segments IV–VII present usually in double rows ……………………………………………… ***Planococcus minor*** (Maskell)- Ratio of lengths of hind tibia + tarsus to trochanter + femur 1.1–1.13. Multilocular disc pores on posterior edges of abdominal segments IV–VII present usually in single rows ………………………………………………………. ***Planococcus citri*** (Risso).

23. Multilocular disc pores present on dorsum in rows across the segments, particularly on abdomen …………………………… 24- Multilocular disc pores on dorsum not present in rows across the segments ……………………………………………………….. 26

24. Numerous dorsal setae each with a few trilocular pores present next to setal collars ……………………………………………………………...…….. ***Phenacoccus madeirensis*** Green- Dorsal setae without trilocular pores next to setal collars, except for possibly for occasional pores ……………………………… 25

25. Dorsal multilocular disc pores present on head. Translucent pores present on hind femora …………………………………………………… ***Phenacoccus gregosus*** Williams & Granara de Willink- Dorsal multilocular disc pores absent from head. Translucent pores absent from hind femora ……… ***Phenacoccus helianthi*** (Cockerell).

26. Quinquelocular pores absent from venter ………..….….... ***Phenacoccus solenopsis*** Tinsley- Quinquelocular pores present on venter …………………………………………………... 27

27. Quinquelocular pores anterior to clypeolabral shield numbering 32–68 ………………… ***Phenacoccus manihoti*** Matile-Ferrero- Quinquelocular pores anterior to clypeolabral shield numbering 0–20 ……….……………………***Phenacoccus herreni*** Cox & Williams.

## Discussion

The potential spread of *P. manihoti* into more Asian countries remains a prime concern for cassava production in the region [6]. In an effort to support decisions to manage this invasive pest, this article reports *P. manihoti*'s known invasive distribution, predicts its potential distribution in Asia, and presents a taxonomic key that distinguishes it from all other mealybug species associated with the genus *Manihot*.

To our knowledge, our article is the first to report *P. manihoti*'s occurrence in Cambodia and Vietnam, suggesting the pest is rapidly spreading in the region. We know of only one additional study predicting *P. manihoti*'s potential distribution, but based on correlative models [47]. This article complements the previous effort by parametrizing a mechanistic model (CLIMEX), using an integrative inductive-deductive model fitting approach. Prediction patterns from both models are very similar, with a tendency of the CLIMEX model to be more conservative (e.g., predicting no suitability where the correlative model predicts low suitability). One important advantage of the CLIMEX model is that it allowed us to formulate specific hypotheses on the climatic factors potentially limiting *P. manihoti*'s spread in Asia. The model is also temporally explicit, and could therefore be instrumental in the design and planning of early detection programs.

Results suggest *P. manihoti* is (1) broadly adapted to the Southeast Asian climates, but is likely to be limited by (2) cold in northern latitudes (>20°N) and (3) high rainfall around the Equator. In ecological terms, our pest risk map represents a hypothesis of what environments in Asia fall within *P. manihoti*'s fundamental niche. The fundamental niche is a concept representing the full range of environmental conditions where a species can survive and reproduce in the absence of negative interactions with other species [48]. Accordingly, the risk map does not take into account the effects of natural enemies and human intervention, among other limiting factors that should further restrict *P. manihoti*'s distribution. In that respect, our study, combined with previous mechanistic modeling work for *P. manihoti* [20], could be used as the basis of a more comprehensive model that also accounts for the potential suppressive role of *A. lopezi* [49].

Our model predictions should be particularly important to guide management decisions for high risk areas where *P. manihoti* is absent (e.g., India), or where it has established but populations remain small and localized (e.g., South Vietnam). For those locations, management options include prevention, eradication and containment (Fig. 6) [50]–[52]. The development of plant quarantine measures to prevent introductions at likely entry pathways is the first and most cost-effective option where a pest is absent [50]–[53]. It can be achieved by intercepting, treating or prohibiting the entry of contaminated or potentially-contaminated material (e.g., cassava planting stakes [54]) [52]. When prevention fails, eradication is the preferred course of action [50]–[53], [55]. Insect eradication can be achieved with insecticide or biopestide treatments designed to eliminate the pest from a delimited area [52], [56]. Finally, containment involves managing the spread of invasion either by reducing dispersal, reducing population growth or a combination of both [51], [56]. Viable containment tactics include domestic quarantines, insecticide treatments and classical biological control at the expanding population front [56]. Successful eradication and containment rest on the ability to detect low-density populations, demanding the development of species-specific surveying methods that are practical and cost effective [52], [53], [56]. In that respect, our model could be used as a tool to design a risk-based surveying program, specific in space and in time, that improves the probability of detecting nascent *P. manihoti* populations.

The window of opportunity for *P. manihoti* early detection and rapid response may be small once the invasion reaches its spread phase. In Africa, the cassava mealybug spread at a rate of 150 km/year [4], contrasting the less than 30 km/year for other invasive Hemiptera [56]. Similarly in Thailand, *P. manihoti* spread widely and began causing yield losses as high as 50%, estimated at roughly US$ 30 million, within two years of first detection [4], [47]. This aggressive spread is poorly explained by the insect's dispersal biology. Instead, anthropogenic mechanisms such as the movement of contaminated planting stakes, where mealybugs can survive feeding on buds [54], are more likely drivers. Based on this hypothesis, we believe promoting the soaking of cassava cuttings on an aqueous solution of thiamethoxam (0.2 g/L), imidacloprid (0.8 g/L) or dinotefuran (8 g/L) may be an effective tactic to slow mealybug spread. Ultimately, however, successful management of *P. manihoti* spread will require a better understanding of the mechanisms contributing to its long-distance dispersal.

In summary, the arrival of *P. manihoti* in Asia imposes an important, yet differential, threat to cassava production in the region. The identification key presented in this article should help qualified experts accurately distinguish it from similar species associated with cassava. Our mechanistic model accurately matched *P. manihoti*'s known distribution and a previous correlative distribution model, suggesting it is good working hypothesis on the mealybug's potential distribution in Asia. This new model, in addition to the recent sightings reported in this article, should help decision-makers assess site-specific risk of invasion, and develop proportional prevention and surveillance programs for early detection and rapid response.
